# Arsenic speciation in beverages by direct injection-ion chromatography hydride generation atomic fluorescence spectrometry

**DOI:** 10.1155/S1463924600000043

**Published:** 2000

**Authors:** E. Moreno, C Cámara, W. T. Corns, D. W. Bryce, P. B. Stockwell

**Affiliations:** 1 University of Complutense Madrid Spain; 2 PS Analytical Arthur House Crayfields Industrial Estate Main Road Orpington Kent BR5 3HP UK

## Abstract

The procedure developed allows the direct speciation of arsenic in these samples with good sensitivity, selectivity, precision and accuracy. Detection limits determined using the optimized conditions were found to be between 0.16 and 2.9ng ml^−1^ for arsenite, dimethylarsinic acid, monomethylarsonic acid and arsenate, while standard addition studies showed that the procedure is free from matrix interferences. As no certified reference materials are available for these analytes or matrices, validation was carried out by studying spike recoveries and by comparison of results with an alternative technique.


Journal of Automated Methods & Management in Chemistry, Vol. 22, No. 2 (March–April 2000) pp. 33–39

### Arsenic speciation in beverages by direct injection-ion chromatography—hydride generation atomic fluorescence spectrometry

**E. Moreno, C. Cámara, W.T. Corns,[1] D.W. Bryce[1] and P.B. Stockwell[1]**

*University of Complutense, Madrid, Spain; [1]PS Analytical, Arthur House, Crayfields Industrial Estate, Main Road, Orpington, Kent BR5 3HP, UK. e-mail: psa@psanalytical.demon.co.uk*

*This paper reports the advantages of coupling strong anion exchange high-performance liquid chromatography hydride generation and atomic fluorescence spectrometry for the speciation of four arsenic species in wine and mineral water.*

*The procedure developed allows the direct speciation of arsenic in these samples with good sensitivity, selectivity, precision and accuracy. Detection limits determined using the optimized conditions were found to be between 0.16 and 2.9 ng ml$^{-1}$ for arsenite, dimethylarsinic acid, monomethylarsonic acid and arsenate, while standard addition studies showed that the procedure is free from matrix interferences. As no certified reference materials are available for these analytes or matrices, validation was carried out by studying spike recoveries and by comparison of results with an alternative technique.*

### Introduction

For many years, it was sufficient for analysts to determine only the total concentration of toxic elements in samples. However, recently it has become apparent that the toxicity, mobility and bioavailability of certain elements depends heavily on their physiochemical form. This is particularly true in the case of arsenic, a well-known toxic element, which may be found in as many as 13 forms [1, 2]. In general, inorganic arsenic (arsenite and arsenate) is more toxic than organic species, e.g. monomethylarsonic acid, dimethylarsinic acid, arsenobetaine, arsenocholine and tetramethylarsonium ion.

Arsenic's presence in the environment is due to both natural and anthropogenic sources. Arsenic is often found in herbicides, pesticides and insecticides (many of which environment protection agencies are now banning) [3], and it is through this route that arsenic gets into wines and other drinks. European legislation has set maximum permissible (total) arsenic concentrations of 1 and 10 ng ml$^{-1}$ in wine and drinking water, respectively. Inorganic arsenic compounds, which are known carcinogens, are used in many manufacturing industries, e.g. glass production, wood preservation and the production of lead accumulators, and are metabolized in the body prior to excretion. Organic arsenic species, which are generally considered to be non-toxic, are often found in fish, seafood and mushrooms.

With the total concentration of arsenic in these samples being so low, and the number of individual forms in which it may be present, it is necessary to develop methods with suitably high sensitivity and selectivity to enable accurate determination of each individual arsenic species.

Methods for speciation have to couple the best of separation with the best detection in order to obtain the necessary sensitivity and selectivity. Atomic spectrometric methods using hydride generation have been used frequently as this particular method of sample introduction reduces many interferences and allows greater sample introduction efficiency, so allowing lower detection limits to be reached. However, most of the reported methods are based on atomic absorption which does not show sufficient sensitivity for low levels of arsenic, thus meaning that pre-concentration steps are necessary [4–7]. Electrothermal atomic absorption (ETAAS) has been used frequently for the determination of arsenic in several types of samples, as the technique shows good sensitivity. However, the technique suffers from serious interference effects, making the use of chemical modifiers necessary. Recent publications in the field make it obvious that hydride generation atomic fluorescence spectrometry and hydride generation ICP-MS are the two techniques of choice for the hydride-forming elements, as they offer the lowest limits of detection [8, 9]. Although both approaches appear to offer similar detection limits, HG/ICP-MS is unsuitable for many laboratories due to high initial and running costs, together with the levels of maintenance required to keep the instrument operating. Hydride generation AFS, on the other hand, is relatively inexpensive and maintenance free, while offering unsurpassed analytical performance in terms of linearity, sensitivity and freedom from interferences [10–13]. Several publications on the determination of total arsenic levels in matrices ranging from sea water [14], wines and beers [15] to hair [16] have appeared in recent years using hydride generation atomic fluorescence.

There is a large body of literature on the speciation of arsenic using ion pair chromatography and anion exchange chromatography [17–22], usually using atomic absorption detection and atomic fluorescence spectrometry. However, it is strange to find that there are very few publications using hydride generation atomic fluorescence spectrometry, and in particular, there are no publications on the speciation of arsenic as As (III), DMA, MMA and As (V) in mineral waters and wines.

The aim of this study was to apply chromatographic separation using a strong anion exchange column, followed by hydride generation atomic fluorescence spectrometry for the determination of As (III), DMA, MMA and As (V) in mineral waters and wines.

### Experimental

### *HPLC system*

A Spectra Physics System 1000 HPLC pump and a six-port injection valve (Part No. 7125, Rheodyne, CA, USA) were used in conjunction with a strong anion exchange column (PRPX-100, 250 × 4.6 mm, 10 μm particle size, Hamilton) to achieve separation of the arsenic species. During the analysis of samples, a guard column (GLC 4-SAX, SGE) was used to preserve the column.

### *Hydride generation*

On-line arsine generation was obtained by use of a peristaltic pump (PS Analytical, Kent, UK), and various mixing coils prepared in 0.5 mm I/D PTFE tubes. The volatile hydrides were separated from other reaction by-products in a gas–liquid separator (PS Analytical). Moisture was removed from the volatile hydrides by passing through a membrane drying tube (Perma Pure Products, Farmingdale, NJ, USA).

### *Detection*

An Excalibur atomic fluorescence spectrometer (PS Analytical), equipped with a boosted discharge hollow cathode lamp (Photron, Victoria, Australia), was used for detection. This system includes a hydrogen diffusion flame as atom cell and optical UV filter with a spectral band pass of 20 nm, so allowing three resonance wavelengths of arsenic to be collected.

### *Data collection*

The fluorescence signal was recorded on a potentiometer chart recorder Servoscribe RE 541.20.

### *Validation*

In order to validate results obtained with the proposed system for the speciation of arsenic, samples were also analysed for total arsenic using a Millennium Excalibur system (PS Analytical). Data collection and treatment was by Avalon software (PS Analytical). Detailed explanations of the instrument are given in previously published papers [10, 16].

### *Reagents*

All reagents were of analytical grade, and de-ionized water was used throughout for the preparation of solutions.

Standard solutions (1000 μg $ml^{-1}$) of arsenite and arsenate were prepared by dissolving 0.1734 g of $NaAsO_2$ and 0.4164 g $Na_2HAsO_4{\cdot}7H_2O$, respectively, in de-ionized water and diluting to 100 ml. MMA and DMA solutions (1000 μg $ml^{-1}$) were prepared by dissolving 0.3894 g of $CH_3AsO(ONa)_2{\cdot}6H_20$ and 0.1840 g of $(CH_3)_2AsHO_2$ in de-ionized water and diluting to 100 ml. Working solutions were made after suitable dilution in the mobile phase. This mobile phase was 10 mM $K_2HPO_4$ and 10 mM $KH_2PO_4$ adjusted to pH 5.7. This was prepared by dissolving 1.74 g $K_2HPO_4$ and 1.36 g $KH_2PO_4$ in ~950 ml $H_2O$ and adjusting the pH by dropwise addition of a 50% HCl solution until pH 5.7 was obtained. This was then diluted to 1000 ml in de-ionized water and degassed by bubbling with helium for 30 min prior to use.

A solution of 1.4% m/v sodium borohydride in 0.1 M sodium hydroxide was used as the reductant and was prepared by firstly dissolving 4.0 g NaOH (BDH Merck) in ~500 ml de-ionized water. Following this, 14.0 g $NaBH_4$ (Aldrich) was added and dissolved before finally diluting the solution to 1000 ml with de-ionized water. This solution was prepared fresh daily.

Hydrochloric acid, potassium iodide and ascorbic acid were all of AnalaR grade (BDH, Merck).

### Procedure

### *Speciation of arsenic*

A schematic diagram of the ion chromatography-hydride generation-atomic fluorescence system used is shown in figure 1. Optimization of the system is explained in detail in a previous paper by Gomez Ariza *et al.* [20].

A portion (200 μl) of standard or sample is introduced via the injection valve into a mobile phase of 10 mM potassium phosphate ($K_2HPO_4/KH_2PO_4$), pH 5.7, flowing at 0.8 ml $min^{-1}$. From here the samples pass onto the strong anion exchange column where the four arsenic species are separated. On elution from the column, the stream is then acidified by mixing with a stream of 1.5 M HCl flowing at 1.5 ml $min^{-1}$. The reagents then pass to a gas–liquid separator where a stream of argon flowing at 250 ml $min^{-1}$ purges the headspace, flushing the volatile hydrides and the hydrogen formed in the reaction through a semi-permeable membrane (which is continuously dried with air flowing in the opposite direction at 2.5 l $min^{-1}$) and to the detector. The hydrogen gas, which is a by-product of the hydride generation reaction, is used as fuel for the hydrogen diffusion flame, which serves to provide free arsenic atoms. These free atoms are then excited by the boosted discharge hollow cathode lamp causing them to fluoresce, the fluorescence being detected by the PMT and converted to a 0–1 V output signal, recorded on a chart recorder. Arsenic species were identified on the basis of retention time. Table 1 summarizes the chromatographic, hydride generation and atomic fluorescence conditions used throughout the study.

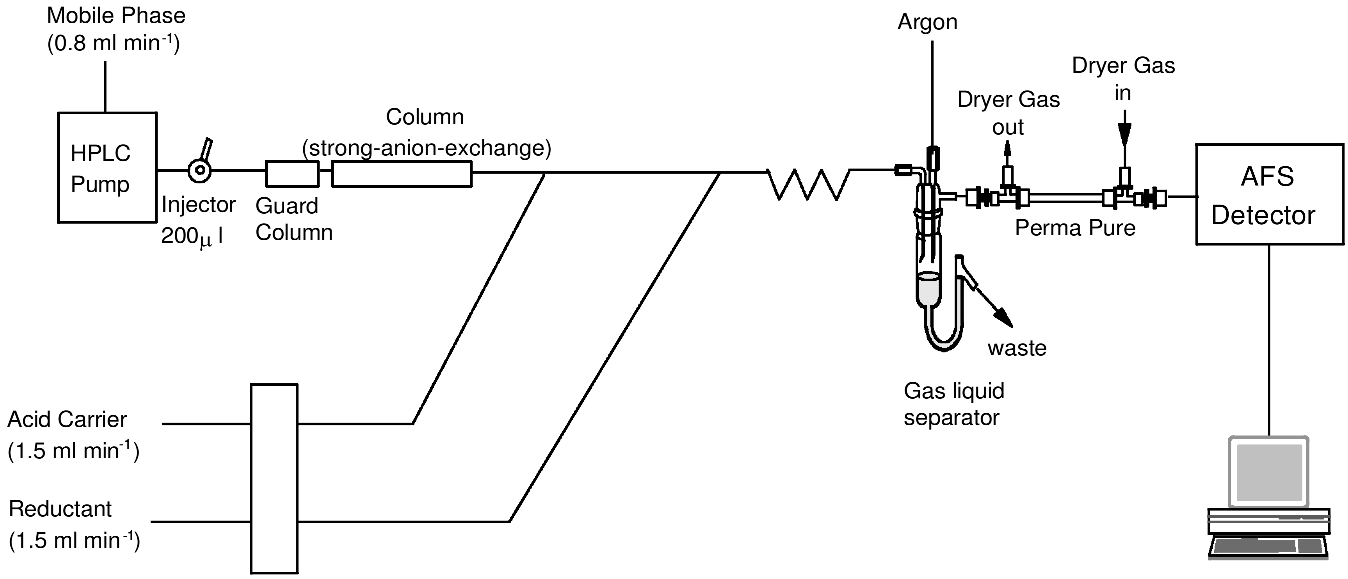

*Figure 1. Schematic diagram of the ion chromatography HG-AFS system for arsenic speciation.*

*Table 1. Instrumental and chemical conditions for the speciation of arsenic using IC-HG-AFS.*

| | |
|---|---|
| Chromatographic conditions | |
| Guard column | GLC 4-SAX (SGE) |
| Column | PRP X-100 (SAX), 250 × 4.6 mm, 10 µm (Hamilton) |
| Mobile phase | 10 mM $K_2HPO_4$, 10 mM $KH_2PO_4$, 0.8 ml min$^{-1}$, pH 5.7 |
| Injected volume | 200 µl |
| Sample | Prepared in mobile phase |
| Hydride generation conditions | |
| Acid solution | 1.5 M HCl, 1.5 ml min$^{-1}$ |
| Reductant | 1.4% m/v $NaBH_4$ in 0.1 M NaOH, 1.5 ml min$^{-1}$ |
| Carrier gas | Argon, 250 ml min$^{-1}$ |
| Dryer gas | Air, 2.5 l min$^{-1}$ |
| Atomic fluorescence conditions | |
| Primary current | 27.5 mA |
| Boost current | 35.0 mA |

*Table 2. Instrumental and chemical conditions for total arsenic using HG-AFS.*

| | |
|---|---|
| Hydride generation conditions | |
| Reagent blank | 25% v/v HCl, 1% m/v KI, 0.2% m/v ascorbic acid, 9.0 ml min$^{-1}$ |
| Reductant | 0.7% m/v $NaBH_4$ in 0.1 M NaOH, 4.5 ml min$^{-1}$ |
| Sample | in 25% v/v HCl, 1% m/v KI, 0.2% m/v ascorbic acid, 9.0 ml min$^{-1}$ |
| Carrier gas | Argon, 235 ml min$^{-1}$ |
| Dryer gas | Air, 2.5 l min$^{-1}$ |
| Atomic fluorescence conditions | |
| Primary current | 27.5 mA |
| Boost current | 35.0 mA |

### *Total arsenic determination*

In order to validate the speciation results, samples were also analysed for total arsenic. In this case, off-line sample pre-treatment by acidification and pre-reduction of all arsenic species to As (III) was carried out. This was achieved by diluting the samples in 25% v/v HCl, 1% m/v KI and 0.2% m/v ascorbic acid and leaving for 30 min. Samples and standards were treated alike. The system used was based on continuous flow hydride generation atomic fluorescence, and as such a reagent blank of 25% v/v HCl, 1% m/v KI and 0.2% m/v ascorbic acid was also prepared. In this case the reductant used was 0.7% m/v $NaBH_4$ in 0.1 M NaOH. Operating conditions are given in table 2.

*Table 3. Performance characteristics of the proposed method for aqueous standards.*

| Species | Concentration ($ng\,ml^{-1}$) | Equation | Correlation coefficient, $r^2$ | LOD[a] ($ng\,ml^{-1}$) | LOQ[b] ($ng\,ml^{-1}$) |
|---|---|---|---|---|---|
| As(III) | 0–10 | $y = 2.049x - 0.06$ | 0.9994 | 0.16 | 0.54 |
| DMA | 0–10 | $y = 0.987x + 0.08$ | 0.9939 | 0.33 | 1.11 |
| MMA | 0–10 | $y = 1.540x + 0.70$ | 0.9969 | 0.32 | 1.08 |
| As(V) | 0–10 | $y = 0.745x + 0.14$ | 0.9965 | 0.57 | 1.90 |

*Table 4. Limits of detection and quantification for various matrices.*

| | Aqueous | | White wine | | Mineral water | |
|---|---|---|---|---|---|---|
| Species | LOD[a] ($ng\,ml^{-1}$) | LOQ[b] ($ng\,ml^{-1}$) | LOD[a] ($ng\,ml^{-1}$) | LOQ[b] ($ng\,ml^{-1}$) | LOD[a] ($ng\,ml^{-1}$) | LOQ[b] ($ng\,ml^{-1}$) |
| As(III) | 0.16 | 0.54 | 0.37 | 1.24 | 0.32 | 1.06 |
| DMA | 0.33 | 1.11 | 0.46 | 1.53 | 0.30 | 1.00 |
| MMA | 0.32 | 1.08 | 0.72 | 2.41 | 0.33 | 1.13 |
| As(V) | 0.57 | 1.90 | 0.87 | 2.90 | 0.47 | 1.57 |

[a]LOD calculated as $3_{\sigma n-1}$ of $2\,ng\,ml^{-1}$ ($n = 10$).

[b]LOQ calculated as $10_{\sigma n-1}$ of $2\,ng\,ml^{-1}$ ($n = 10$).

### Results and discussion

### *Characteristics of the proposed method*

For the purpose of this study, the proposed system was calibrated from 0 to $10\,ng\,ml^{-1}$ As (III), DMA, MMA and As (V). Typical equations of calibration curves and correlation coefficients are given in table 3, while limits of detection and quantification in various sample matrices are shown in table 4. These limits were calculated as three and 10 times the standard deviation of 10 runs of a $2\,ng\,ml^{-1}$ standard, respectively. Results show that the best sensitivity was shown for As(III) followed by MMA, DMA and finally As(V). Although this is partially related to the chromatography, it is mainly due to the hydride generation step where it is well known that As(III) forms a hydride more efficiently than As(V).

A typical chromatogram, obtained using the conditions outlined in table 1, for a mixed solution of $2.5\,ng\,ml^{-1}$ As (III), DMA, MMA and As (V) is shown in figure 2. The retention times for each species were found to be 2.25, 3.20, 5.20 and 9.15 min for As (III), DMA, MMA and As (V), respectively. These retention times were used to identify arsenic species in unknown samples.

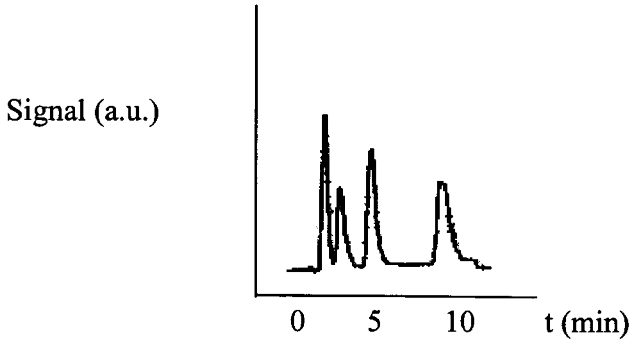

*Figure 2. Chromatogram under optimum conditions for a mixed 2.5 ng ml$^{-1}$ standard.*

The precision of the proposed method was studied by carrying out repeated injections ($n = 10$) of a mixed $2\,ng\,ml^{-1}$ standard containing As (III), DMA, MMA and As (V). Precision was studied not only in aqueous standards, but also in sample matrix, i.e. white wine and mineral water. Table 5 summarizes the results, which show that overall the best precision (expressed as per cent relative standard deviation, RSD) is found for As(III), with RSDs ranging from 1.35% in white wine to 4.20% in mineral water. The best precision was found for As(III) in white wine, although this is probably due to the fact that this was due to the As(III) found in the sample (i.e. not a spiked concentration) which was actually $5.51\,ng\,ml^{-1}$ instead of $2\,ng\,ml^{-1}$ which was the concentration studied for all other species. The results also show that the precision for As(V) is also notably lower in the mineral water than in white wine or the aqueous standard, again probably due to the fact that the As(V) in the mineral water was present at $15.40\,ng\,ml^{-1}$ as opposed to $2\,ng\,ml^{-1}$. Overall, however, the precision ranges from 1.35 to 8.75%.

### *Applications of the proposed method*

The proposed method was applied to the determination of arsenic species in wines and mineral waters. Figure 3 shows typical chromatograms for various samples, showing that the only species present in the samples were As(III) and As(V). Figure 3 shows that As(III) was found in all the wine samples tested but none of the mineral water samples. As(V), however, was found in only one of the white and one of the red wines. More surprisingly it was found at a relatively high concentration ($15.41\,ng\,ml^{-1}$) in one of the mineral water samples (French) but not in the other (Scottish). When analysing

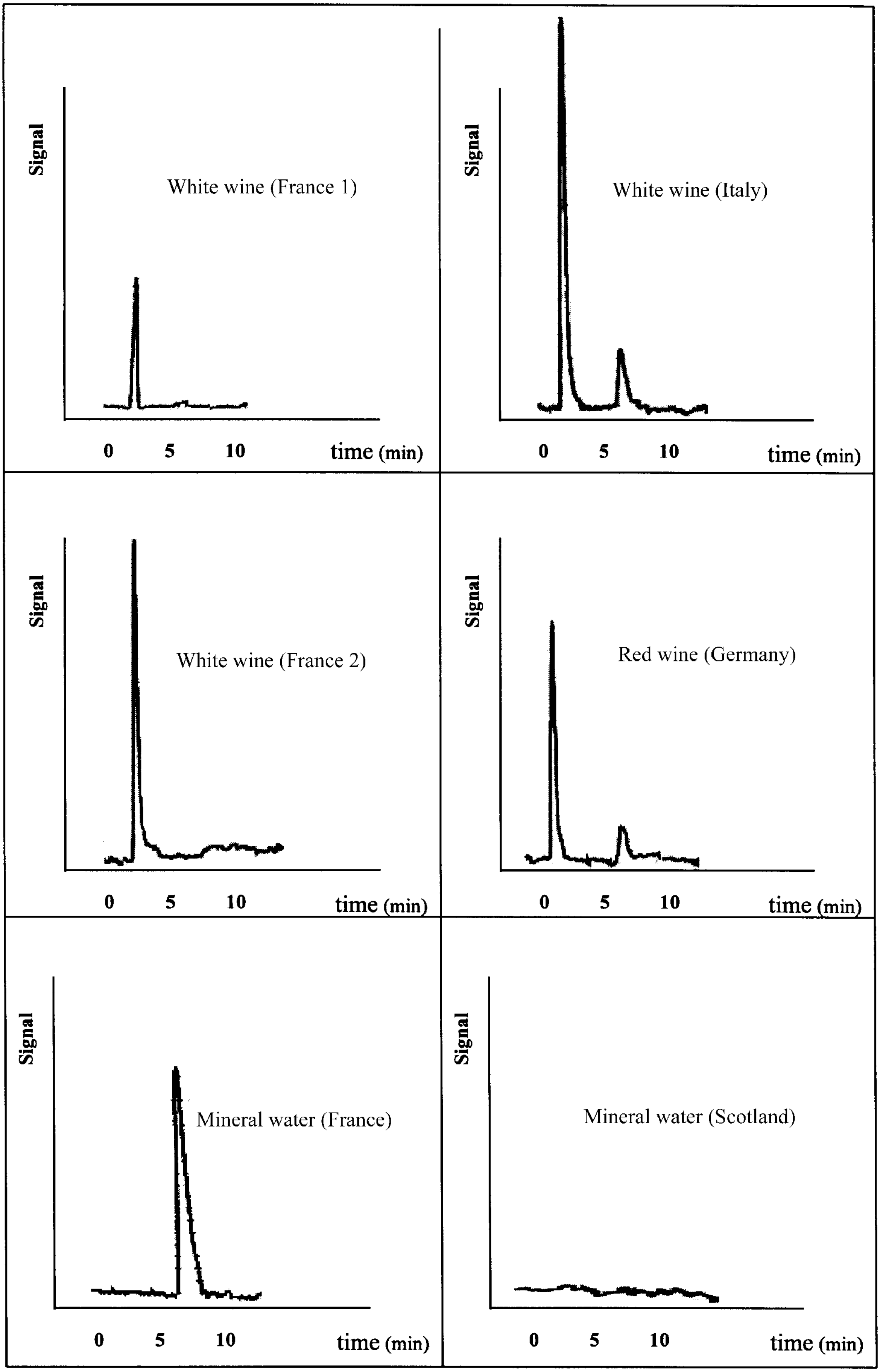

*Figure 3. Chromatograms for wine and water samples.*

*Table 5. Precision for arsenic species in different samples.*

| | Precision (% RSD)[a] | | | |
|---|---|---|---|---|
| Sample | As(III) | DMA | MMA | As(V) |
| Aqueous | 2.39 | 5.19 | 5.18 | 7.75 |
| Mineral water (France) [15.41 $ng\,ml^{-1}$ As(V)] | 4.20 | 2.37 | 4.50 | 1.95 |
| White wine (France 1) [5.51 $ng\,ml^{-1}$ As(III)] | 1.35 | 7.77 | 8.75 | 6.36 |

[a] Percentage relative standard deviation based on 10 runs of 2 $ng\,ml^{-1}$ spikes (unless stated otherwise).

*Table 6. Results for real samples.*

| | Concentration found ($ng\,ml^{-1}$) | | | | | |
|---|---|---|---|---|---|---|
| Sample | As(III) | DMA | MMA | As(V) | Total[a] | As total by comparison[b] |
| White wine (France 1) | 5.36 ± 0.24 | ND | ND | ND | 5.36 ± 0.24 | 5.04 ± 0.25 |
| White wine (Italy) | 12.18 ± 0.26 | ND | ND | 3.88 ± 0.28 | 16.06 ± 0.54 | 16.17 ± 0.01 |
| White wine (France 2) | 8.77 ± 0.12 | ND | ND | ND | 8.77 ± 0.12 | 8.44 ± 0.03 |
| Red wine (Germany) | 9.07 ± 0.32 | ND | ND | 1.41 ± 0.20 | 10.48 ± 0.52 | 9.50 ± 0.05 |
| Mineral water (France) | ND | ND | ND | 15.40 ± 0.28 | 15.40 ± 0.28 | 14.80 ± 0.06 |
| Mineral water (Scotland) | ND | ND | ND | ND | ND | ND |

ND, not detected.

[a] Calculated as the sum of the individual species, identified on the basis of retention time.

[b] Determined using the Millennium Excalibur system for total arsenic.

*Table 7. Results for spike recoveries.*

| | Spike recoveries ($ng\,ml^{-1}$) | | | | | | | |
|---|---|---|---|---|---|---|---|---|
| | As(III) | | DMA | | MMA | | As(V) | |
| Sample | Added | Found | Added | Found | Added | Found | Added | Found |
| White wine (France 1) | 2.5 | 2.65 ± 0.18 | 2.5 | 2.20 ± 0.16 | 2.5 | 2.67 ± 0.24 | 2.5 | 2.02 ± 0.28 |
| | 5.0 | 4.87 ± 0.19 | 5.0 | 5.35 ± 0.15 | 5.0 | 4.91 ± 0.22 | 5.0 | 5.37 ± 0.27 |
| | 10.0 | 10.03 ± 0.18 | 10.0 | 9.92 ± 0.17 | 10.0 | 10.04 ± 0.19 | 10.0 | 9.91 ± 0.20 |
| | 20.0 | 20.25 ± 0.22 | 20.0 | 19.61 ± 0.11 | 20.0 | 20.40 ± 0.17 | 20.0 | 19.83 ± 0.12 |
| White wine (Italy) | 2.5 | 2.38 ± 0.06 | 2.5 | 2.69 ± 0.10 | 2.5 | 2.69 ± 0.09 | 2.5 | 2.40 ± 0.58 |
| | 5.0 | 5.06 ± 0.03 | 5.0 | 4.91 ± 0.12 | 5.0 | 4.89 ± 0.11 | 5.0 | 5.05 ± 0.09 |
| | 7.0 | 6.56 ± 0.17 | | | | | 7.0 | 6.90 ± 0.03 |
| White wine (France 2) | 2.5 | 2.33 ± 0.23 | 2.5 | 2.68 ± 0.25 | 2.5 | 2.75 ± 0.13 | 2.5 | 2.40 ± 0.07 |
| | 5.0 | 4.99 ± 0.06 | 5.0 | 4.98 ± 0.15 | 5.0 | 4.92 ± 0.12 | 5.0 | 5.07 ± 0.01 |
| Red wine (Germany) | 2.5 | 2.42 ± 0.15 | 2.5 | 2.30 ± 0.26 | 2.5 | 2.58 ± 0.12 | 2.5 | 2.46 ± 0.13 |
| | 5.0 | 5.03 ± 0.02 | 5.0 | 4.98 ± 0.14 | 5.0 | 4.92 ± 0.11 | 5.0 | 5.07 ± 0.02 |
| Mineral water (France) | 2.5 | 2.50 ± 0.02 | 2.5 | 2.90 ± 0.06 | 2.5 | 2.50 ± 0.11 | 2.5 | 2.48 ± 0.04 |
| | 5.0 | 5.00 ± 0.02 | 5.0 | 5.08 ± 0.11 | 5.0 | 4.95 ± 0.07 | 5.0 | 5.00 ± 0.06 |

the samples, two- and fivefold dilutions were carried out on each sample, in order to detect the presence of matrix interferences. No matrix effects were observed. Results for the concentrations of the different species found are shown in table 6.

No wine or water reference materials with certified arsenic species are currently available, and so in order to validate the method, two approaches were used. Firstly, samples were spiked with known concentrations of each of the arsenic species and the recoveries calculated. Secondly, samples were analysed for total arsenic using an alternative technique.

All samples were spiked with both 2.5 and 5 $ng\,ml^{-1}$ of each of the four arsenic species, and some with 7.0, 10.0

and 20.0 ng $ml^{-1}$. The spike recoveries, shown in table 7, show quantitative recoveries of all species, showing that the method is free from interferences.

For analysis of total arsenic, a PSA 10.055 Millennium Excalibur system (PS Analytical) was used. This system, which has been described elsewhere [10, 16], is based on hydride generation atomic fluorescence. Samples must first be acidified to 25% v/v HCl, following which KI and ascorbic acid must be added in order to convert all arsenic in the sample to As (III) to facilitate hydride generation. Results for total arsenic concentrations are also given in table 6. In all cases good agreement is observed with the results using the proposed IC-HG-AFS system.

### Conclusions

Atomic fluorescence is an extremely sensitive detection system for arsenic which can be easily coupled to an ion chromatography system, allowing the determination of individual arsenic species. The results reported here show that the proposed method is accurate and sensitive enough to carry out arsenic speciation in wines and mineral water. In addition, the method shows high selectivity, needs no sample pre-treatment and is free from interferences.

### Acknowledgement

The authors wish to thank the CICYT project PB95-0366-CO1-CO2 for financial support.

### References

1. Cullen, W. R. and Reimer, K. J., *Chem. Rev.*, **89** (1989) 713.
2. Andreae, M. D., in P. J. Craig (ed.) *Organometallic Compounds in the Environment* Longman, London (1986).
3. Segura, M., Madrid, Y. and Camara, C. (in press).
4. Agterdembos, J. and Juari, S., *Anal. Chim. Acta*, **237** (1990) 189.
5. Fag, Z., Xu, S. and Tao, G., *J. Anal. At. Spectrometry*, **11** (1996) 1.
6. Amankakwash, S. A. and Fasching, J. L., *Talanta*, **32** (1985) 111.
7. Nakashima, S., *Fres. J. Anal. Chem.*, **341** (1991) 370.
8. Story, W. C., Caruso, J. A., Heitkemper, O. T. and Perkins, L., *J. Chromatogr. Sci.*, **30** (1992) 427.
9. Lee, X. C., Ma, M. and Wong, N. A., *Anal. Chem.*, **68** (1996) 4501.
10. Stockwell, P. B. and Corns, W. T., *Analyst*, **119** (1994) 1641.
11. Stockwell, P. B. and Corns, W. T., *International Labmate*, **33**.
12. Corns, W. T., Stockwell, P. B., Ebdon, L. and Hill, S. J, *J. Anal. At. Spectometry*, **11** (1996) 61.
13. Corns, W. T., Ebdon, L., Hill, S. J. and Stockwell, P. B., *Analyst*, **117** (1992) 717.
14. Moreda-Pineiro, J., Cervera, M. L. and de la Guardia, M., *J. Anal. At. Spectrometry*, **12** (1997) 1377.
15. Madrid, Y., Segura, M. and Camara, C. (in press).
16. Rahman, L., Corns, W. T., Bryce, D. W. and Stockwell, P. B., *Talanta*, in press.
17. Spall, W. D., Lynn, J. G., Andersen, J. L., Valdez, J. G. and Gurley, L. R., *Anal. Chem.*, **58** (1986) 1340.
18. Thomson, J. J. and Houk, R. S., *Anal. Chem.*, **58** (1986) 2541.
19. Morin, P., Amran, M. B., Lakkil, M. D. and Leroy, M. J., *Chromatographia*, 33 (1992) 581.
20. Gomez-Ariza, J. L., Sanchez-Rodas, D., Beltran, R., Corns, W. T. and Stockwell, P. B., *Applied Organometallic Chemistry*, **12** (1998) 439.
21. Van Elteren, J. T. and Slejkovel, Z., *J. Chromatography*, **789** (1997) 339.
22. Mester, Z. and Fodor, P., *J. Chromatography*, **756** (1996) 292.

